# Economic and health impact modelling of a whole genome sequencing-led intervention strategy for bacterial healthcare-associated infections for England and for the USA

**DOI:** 10.1099/mgen.0.001087

**Published:** 2023-08-09

**Authors:** John M. Fox, Nigel J. Saunders, Susie H. Jerwood

**Affiliations:** ^1^​ Genpax, London, UK

**Keywords:** healthcare-associated infections, genomics, bacteria, diagnostics, economics

## Abstract

Bacterial healthcare-associated infections (HAIs) are a substantial source of global morbidity and mortality. The estimated cost associated with HAIs ranges from $35 to $45 billion in the USA alone. The costs and accessibility of whole genome sequencing (WGS) of bacteria and the lack of sufficiently accurate, high-resolution, scalable and accessible analysis for strain identification are being addressed. Thus, it is timely to determine the economic viability and impact of routine diagnostic bacterial genomics. The aim of this study was to model the economic impact of a WGS surveillance system that proactively detects and directs interventions for nosocomial infections and outbreaks compared to the current standard of care, without WGS. Using a synthesis of published models, inputs from national statistics, and peer-reviewed articles, the economic impacts of conducting a WGS-led surveillance system addressing the 11 most common nosocomial pathogen groups in England and the USA were modelled. This was followed by a series of sensitivity analyses. England was used to establish the baseline model because of the greater availability of underpinning data, and this was then modified using USA-specific parameters where available. The model for the NHS in England shows bacterial HAIs currently cost the NHS around £3 billion. WGS-based surveillance delivery is predicted to cost £61.1 million associated with the prevention of 74 408 HAIs and 1257 deaths. The net cost saving was £478.3 million, of which £65.8 million were from directly incurred savings (antibiotics, consumables, etc.) and £412.5 million from opportunity cost savings due to re-allocation of hospital beds and healthcare professionals. The USA model indicates that the bacterial HAI care baseline costs are around $18.3 billion. WGS surveillance costs $169.2 million, and resulted in a net saving of ca.$3.2 billion, while preventing 169 260 HAIs and 4862 deaths. From a ‘return on investment’ perspective, the model predicts a return to the hospitals of £7.83 per £1 invested in diagnostic WGS in the UK, and US$18.74 per $1 in the USA. Sensitivity analyses show that substantial savings are retained when inputs to the model are varied within a wide range of upper and lower limits. Modelling a proactive WGS system addressing HAI pathogens shows significant improvement in morbidity and mortality while simultaneously achieving substantial savings to healthcare facilities that more than offset the cost of implementing diagnostic genomics surveillance.

## Data Summary

All data and mathematical models have been provided within the article or in the Supplementary File, available in the online version of this article.

**Impact Statement**

This article estimates the impact of effective whole genome sequencing-based surveillance for tracking and intervening in bacterial nosocomial outbreaks of the 11 most common healtcare-associated infection (HAI) species in both England and the USA. The projected outcome would be to reduce the bacterial morbidity and mortality of HAI in hospitals while simultaneously reducing the cost of patient care and increasing the wider cost savings of England and the USA by £478.3 million and $3.2 billion respectively, with more efficient use of hospital resources.

## Introduction

Bacterial healthcare-associated infections (HAIs) are a substantial source of morbidity and mortality worldwide [1, 2], resulting in increased length of hospital stay and high healthcare costs [3]. Whole genome sequencing (WGS) has been promoted as a replacement for standard epidemiological investigation and as a new gold standard for outbreak detection [4]. In a clinical setting, potential transmission events are identified from the phylogenetic relationship between isolates by identifying clusters of isolates within a pre-defined threshold of SNPs [5]. To date, widespread adoption is limited, and most studies have been performed in academic centres with specialist interests, supporting resources, and local bioinformatics analysis infrastructure and expertise. In many other settings both the analysis of sequencing results [6] and the upfront cost of implementation [7] have been identified as obstacles to adoption.

Previous models highlighting the impact of WGS on bacterial HAIs have predicted a wide range of clinical and financial impacts [8–11]. One limitation that partially accounts for their varying conclusions is that they differ in what is addressed, and are relatively narrow in scope [12]. Some studies model the impact of several organisms and their resistant forms across a healthcare region [9], while others detail the reduction of one particularly resistant organism and its impact in a single hospital [13] or country [14]. Methodologies have also differed, including complex artificial intelligence simulations [15] and relatively simple counting [10]. Although the methodologies vary, the consistent conclusion is that a successfully implemented WGS surveillance system would be cost-effective.

HAI economic analyses measure the impacts of successfully modifying infection control programmes in terms of economic savings and patient benefits that result from reducing infections compared to the additional costs incurred from implementing new strategies [16]. This approach has been used in previously published WGS model-based clinical and financial analyses [9, 10]. The paper integral to this work from Gordon *et al*. modelled the six primary drug-resistant nosocomial organisms in Queensland, Australia [9]; by contrast, the model in this current study addressed all estimated bacterial HAIs (sensitive and resistant) from these common pathogen groups as well as additional causes of nosocomial infections, and used more clearly defined and recent estimates of costs and savings.

Accurate estimates of the cost of a newly implemented infection control strategy are central to the analyses. It is also important that accurate and up-to-date information is used as model inputs. The models presented in this paper therefore make use of information from recent implementation studies that include consumables, sequencer rental and staffing costs for all steps from the point at which the process becomes additional to routine diagnostic practice. Similarly, they include the full costs of analysis that would be incurred using an openly accessible commercial analysis system, to the point of return of actionable reports to the clinical teams. National statistics available in England are currently the most robust source of information upon which to base clinical and financial models; the United Kingdom Health Security Agency (UKHSA), and previously as Public Health England, provides organism-specific data which were used in this economic analysis [17, 18].

The aim of building this model was to evaluate the clinical and economic impact of a prospective WGS-led track and trace system of 11 common healthcare-associated and antimicrobial resistance (AMR) priority bacterial pathogens in England and the USA compared to the current standard of care, without WGS.

## Methods

Clinical impact models were created to address the most common nosocomial infections found in the England National Health Service (NHS) and the USA. These are caused by *Staphylococcus aureus, Escherichia coli, Enterococcus* species*, Klebsiella* species*, Enterobacter* species*, Acinetobacter* species*, Stenotrophomonas maltophilia, Clostridioides difficile, Pseudomonas* species (mainly *P. aeruginosa), Citrobacter* species and *Serratia* species (Table 1). The models were built following guidelines set out by both Graves [16] and Sullivan [19] and included information and structure from the works of Gordon [9] and Kumar [10]. Briefly, Graves’s work recommends avoiding high estimates of costs designed to push decision-makers to act and advises caution around length of stay calculations (as some patients are pre-disposed to long stays regardless of their HAI). Sullivan’s paper recommends accounting for the relevant features of the healthcare system, developing a relatively simple cost evaluation calculator, reporting detailed input parameter values and presenting the outcomes in a format accessible to its intended readership. As previously mentioned, the Gordon study was integral to the present work and aided in developing the Excel model and overall structure of the manuscript. Kumar’s work included a previously published economic evaluation, and was used primarily to measure the cost of healthcare workers from the amount of time they would spend on infection prevention and control.

**Table 1.** Model input variables used to estimate the number of hospitalized patients affected by HAIs and colonizations in England and the USA from current practice and from a WGS-based system

Variable	England estimate (lower and upper)	Source	USA estimate (lower and upper)	Source
Population	55980000	[59]	329500000	[60]
Hospital admissions/inpatients	13 800000 (11100000–16500000)	[21, 61]	33 356 853 (30556853–36200000)	[48, 62]
HAI prevalence among admissions (%)	4.7 (2.2–6.9)	[21, 61]	5.05 (4.6–9.3)	[23]
Healthcare professionals infected with HAIs	13900	[21]	n/a	n/a
HAIs	653000	[21]	n/a	n/a
HAIs (total including specialist hospitals)	834000	[21]	1.7 million	[23]
**Infection rate by pathogen (% of total HAIs)**				
* Staphylococcus aureus *	12 (10.2–17.3)	[9, 63]	12 (10.2–17.3)	[9, 63]
* Stenotrophomonas maltophilia *	0.22 (0.07–0.36)	[35]	0.22 (0.07–0.36)	[35]
* Escherichia coli *	8.82 (5.9–11.7)	[9]	8.82 (5.9–11.7)	[9]
* Enterococcus* spp.	7.4 (3.4–8.2)	[9, 64]	7.4 (3.4–8.2)	[9, 64]
* Klebsiella* spp.	7 (2.3–8)	[9, 65]	7 (2.3–8)	[9, 65]
* Enterobacter* spp.	1.93 (0.5–3.3)	[9]	1.93 (0.5–3.3)	[9]
* Acinetobacter* spp.	1.1 (0–2.2)	[9]	1.1 (0–2.2)	[9]
* Clostridioides difficile *	5 (2–5.5)	[21, 66, 67]	10 (8.6*–11.4)	[21, 66, 68]
* Pseudomonas* spp.	1 (0–7)	[42, 69, 70]	3 (0–7)	[42, 69, 70]
* Citrobacter* spp.	1.7 (0.55–3)	[10, 71]	1.7 (0.55–3)	[10, 71]
*Serratia spp*.	1 (0–2)	[72]	1 (0–2)	[72]
**Colonizations:infections**				
MRSA	0.40	[9, 27]	0.40	[9, 27]
* Escherichia coli* (ESBL and CPE)	0.21	[9, 28]	0.21	[9, 28]
VRE	0.24	[29]	0.24	[29]
* Klebsiella* spp. (ESBL and CPE)	0.10	[9, 28]	0.10	[9, 28]
* Enterobacter* spp. (ESBL and CPE)	0.05	[9]	0.05	[9]
* Acinetobacter* spp. (CRAB)	0.08	[9]	0.08	[9]
**Cluster frequency**				
* Staphylococcus aureus *	0.02		0.02	
* Stenotrophomonas maltophilia *	0.02		0.02	
* Escherichia coli *	0.02		0.02	
* Enterococcus* spp.	0.05		0.05	
* Klebsiella* spp.	0.02	[9]	0.02	[9]
* Enterobacter* spp.	0.06		0.06	
* Acinetobacter* spp.	0.06		0.06	
* Clostridioides difficile *	0.03		0.03	
* Pseudomonas* spp.	0.02		0.02	
* Citrobacter* spp.	0.02		0.02	
* Serratia* spp.	0.02		0.02	
**Decreased cluster size**				
* Staphylococcus aureus *	5.38 (1.37–9.38)		5.38 (1.37–9.38)	
* Stenotrophomonas maltophilia *	4.72 (1–9.88)*		4.72 (1–9.88)*	
* Escherichia coli *	10.25 (2.94–17.56)		10.25 (2.94–17.56)	
* Enterococcus* spp.	8.29 (3.89–12.68)		8.29(3.89–12.68)	
* Klebsiella* spp.	3.25 (1.23–5.27)	[9, 34]	3.25 (1.23–5.27)	[9, 34]
* Enterobacter* spp.	6.33 (1–13.87)		6.33 (1–13.87)	
* Acinetobacter* spp.	4 (1–9.88)		4 (1–9.88)	
* Clostridioides difficile *	6.25 (1–13.87)*		6.25 (1–13.87)*	
* Pseudomonas* spp.	4.72 (1–9.88)*		4.72 (1–9.88)*	
* Citrobacter* spp.	4.72 (1–9.88)*		4.72 (1–9.88)*	
* Serratia* spp.	4.72 (1–9.88)*		4.72 (1–9.88)*	

The lower and upper input values refer to the lower and upper limits of available evidence for each variable used as the inputs in the sensitivity analyses. CPE, carbapenemase-producing *Enterobacteriaceae*; ESBL, extended-spectrumbeta-lactamase; MRSA, methicillin-resistant *Staphylococcus aureus*.

*Estimated sensitivity range input based on other pathogen groups.

**Table 2.** Variables used to estimate the cost of WGS, hospital stay and antibiotic treatment for England and the USA

Variable	England (lower and upper input)	Source	USA (lower and upper input)	Source
WGS (total per isolate)	£160 (£115–£203)	[13, 40]	$194 ($138–$245)	[13, 40]
Cleaning and nurse time (per detection)	£61.06 (£51–£89)	[9, 37]	$74 ($61–$107)	[9, 37]
PPE (per day)	£29.38 (£19.77–£36.71)	[9]	$35 ($23.88–$44.35)	[9]
Closed-bed day cost (general ward)	£586 (£175.11–£863.35)	[3, 21]	$1772 ($1735–$2018)	[73]
Closed-bed day cost (ICU)	£1621.16 (£1326–£2,491)	[21, 74]	$2902 ($2644–$3334)	[73]
Cost of consumables (per infection)	£160.50	[37]	$194	[37]
Cost of infection prevention and control team (per infection)	£102.34	[43–49]	£111.35	[10, 43]
**Infection length of stay**				
* Staphylococcus aureus *	20 (4.5–77.2)	[3, 9, 75]	20 (4.5–77.2)	[3, 9, 75]
* Stenotrophomonas maltophilia *	28 (16.99–75)	[76]	28 (16.99–75)	[76]
* Escherichia coli *	26.5 (3.87–64)	[9, 14]	26.5 (3.87–64)	[9, 14]
* Enterococcus* spp.	28 (6–38.4)	[9, 77]	28 (6–38.4)	[9, 77]
* Klebsiella* spp.	20 (2–64)	[9, 57]	20 (2–64)	[9, 57]
* Enterobacter* spp.	22 (8–57.5)	[9, 78, 79]	22 (8–57.5)	[9, 78, 79]
* Acinetobacter* spp.	21.5 (5.8–42.8)	[9]	21.5 (5.8–42.8)	[9]
* Clostridioides difficile *	25 (2–30)	[80, 81]	25 (2–30)	[80, 81]
* Pseudomonas* spp.	21 (1–76.3)	[82]	21 (1–76.3)	[82]
* Citrobacter* spp.	18 (6–23)	[83, 84]	18 (6–23)	[83, 84]
* Serratia* spp.	14 (4–65)	[85, 86]	14 (4–65)	[85, 86]
**Colonization length of stay**				
* Escherichia coli* (CPE)	11 (8–31)	[9]	11 (8–31)	[9]
* Klebsiella* spp.(CPE)	7 (7–31)	[9]	7 (7–31)	[9]
* Enterobacter* spp. (CPE)	7 (3–21)	[9]	7 (3–21)	[9]
* Acinetobacter* spp. (CRAB)	7 (6 -22)	[9]	7 (6 -22)	[9]
**Closed bed days from extra length of hospital stay**				
* Staphylococcus aureus *	9.8 (4.5–77.2)	[3, 9, 75]	9.8 (4.5–77.2)	[3, 9, 75]
* Stenotrophomonas maltophilia *	13 (13–75.01)	[76]	13 (13–75.01)	[76]
* Escherichia coli *	12 (3.87–64)	[9, 14]	12 (3.87–64)	[9, 14]
* Enterococcus* spp.	8 (6–38.4)	[9, 77]	8 (6–38.4)	[9, 77]
* Klebsiella* spp.	10 (2–64)	[9, 57]	10 (2–64)	[9, 57]
* Enterobacter* spp.	10 (8–57.5)	[9, 78, 79]	10 (8–57.5)	[9, 78, 79]
* Acinetobacter* spp.	8 (5.8–42.8)	[9]	8 (5.8–42.8)	[9]
* Clostridioides difficile *	9 (2–30)	[80, 81]	9 (2–30)	[80, 81]
* Pseudomonas* spp.	8 (1–76.3)	[82]	8 (1–76.3)	[82]
* Citrobacter* spp.	7 (6–23)	[83, 84]	7 (6–23)	[83, 84]
* Serratia* spp.	6 (4–65)	[85, 86]	6 (4–65)	[85, 86]
**Cost of antibiotic treatment per infected patient**				
Nafcillin/flucloxacillin IV (MSSA bacteraemia)	£336.00		$510.72	
Nafcillin/flucloxacillin IV (MSSA wound infection)	£168.00		$255.36	
Vancomycin (MRSA bacteraemia)	£315.00		$125.16	
Ampicillin/amoxicillin	£14.85		$33.39	
Vancomycin IV (* Enterococcus faecium* bacteraemia)	£157.50		$62.58	
Linezolid/daptomycin/tigecycline (VRE)	£483.33		$212.8	
Ampicillin/sulbactam IV or co-amoxiclav IV then co-amoxiclav PO	£10.74	[38]	$33.39	[39]
Piperacillin/tazobactam (co-amox resistant)	£270.90		$140.57	
Ertapenem (tazobactam resistant)	£221.55		$292.25	
Meropenem (rrtapenem resistant)	£427.98		$148.68	
Caftazidime-avibactam (carbapenem resistant/CPE)	£1799.70		$7954.38	
Colistin	£308.56		$193.41	
Piperacillin/tazobactam [* Pseudomonas aeruginosa* bacteraemia (sensitive)]	£270.90		$140.57	
Ciprofloxacin PO (Pen allergy)	£11.55		$39.48	
Fidaxomicin (* Clostridioides difficile *)	£1351.00		n/a	
Vancomycin po (* Clostridioides difficile *)	£7552.00		$9870	
Cotrimoxazole	£131.60		$160.33	

CPE, carbapenemase-producing *Enterobacteriaceae*; CRAB, carbapenamase-resistant *Acinetobacter baumanni*; MSSA, methicillin-susceptible *Staphylococcus aureus*; VRE, vancomycin-resistant enterococci.

The model inputs are intentionally conservative, especially where the data from publications and official documents are sparse, to prevent overestimating savings. It also does not include rare but highly expensive impacts of failure to detect and control HAIs, including ward and intensive care unit (ICU) closures, or ward refurbishment or rebuilds [20]; nor the potential litigation costs associated with avoidable HAIs. Sensitivity analyses were carried out to determine the robustness of the impacts of the intervention. The baseline model was established with inputs from a previous study that modelled all HAI costs in England [21]. This is a model generated by a professional organizational change company, working with four different clinical laboratories. In addition, input data from UKHSA were used for all 11 of the pathogen groups modelled [18, 22].

Equivalent public health data are not available in the USA with the exception of Centers for Disease Control (CDC) information on healthcare-associated *C. difficile* and methicillin-resistant *Staphylococcus aureus* (MRSA) [23]. Where there was an absence of species-specific information assumptions based on peer reviewed articles and UK-specific inputs were used to calculate the infection and death rate for the USA HAI clinical model.

## Modelling

All models were constructed and analyses performed in Excel. In addition, inputs for estimating costs were up to date, with references from 2019 to the present day.

The economic analyses were broken up into two sections. The first section addresses the impact of a sequencing intervention on direct hospital resources [personal protective equipment (PPE), consumables, antibiotics and WGS]. The second section addresses the impact of the allocation of healthcare professionals and hospital infrastructure before and after the WGS intervention. The individual models can be accessed in the Supplementary File and are categorized by region. The costs of both direct hospital resources and allocation of healthcare professionals and infrastructure are shown in Table 4. The Supplementary File includes a combined overall difference and Return on Investment calculated beneath the economic model sections in rows 223, 224 and rows 217, 218 for the England model and the USA model respectively.

**Fig. 1.**
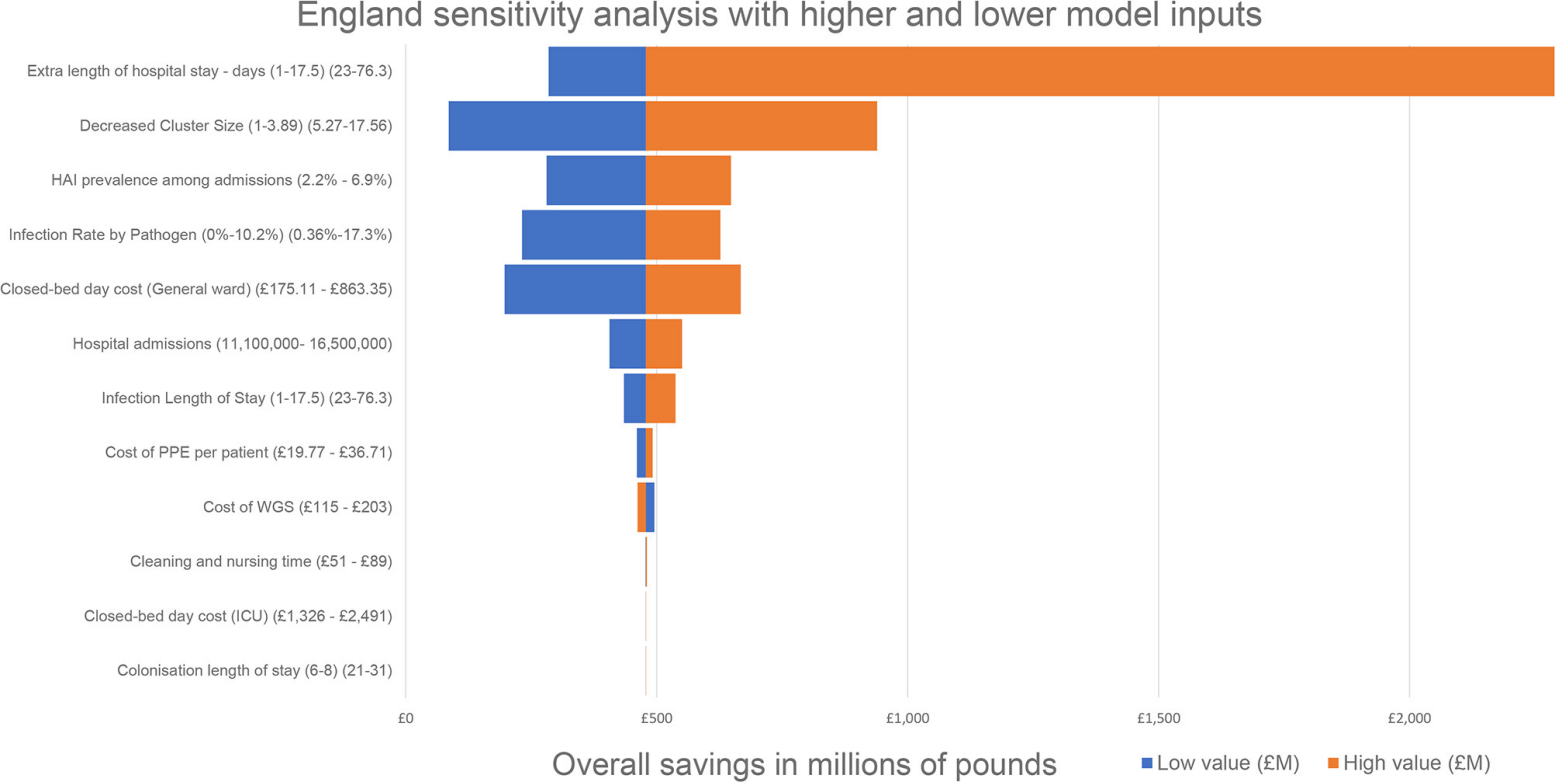
All savings are in millions of pounds relative to a base saving of £478.3 million. Low value (£M) represents the lower input for each variable and equally the High value (£M) represents the higher model input. The higher value for Cost of WGS was the only variable to reduce savings.

Sensitivity analyses were conducted separately for each variable by varying a wide range of upper and lower limits for each variable where evidence of a range of values exists (Tables 1 and 2), and results are provided in Fig. 1 and the Supplementary File (Table S1, Fig. S1, England Model and the USA Model).

## Estimated number of patients developing bacterial HAI

The models presented here generate the estimated number of admissions/inpatients as a percentage of the overall population and, subsequently, the prevalence of all HAIs among hospital admissions/inpatients by working backwards based on evidence from both Guest’s [21] and Haque’s [24] work on HAIs in England and the USA. Each of the pathogen groups modelled account for a proportion of the total number of HAIs; the number of infections was therefore divided accordingly based on different publications estimating the frequency of each pathogen group as a proportion of the total HAIs (Table 1). The relative frequency of each pathogen group can be seen in the Infection Rate by Pathogen section in Table 1. The resistance phenotype [such as MRSA, vancomycin-resistant *Enterococci* (VRE), carbapenemase-producing *Enterobacteriaceae* (CPE), extended-spectrum beta-lactamases (ESBL) and carbapenamase-resistant *Acinetobacter baumannii* (CRAB)], specific species type and bacteraemia/bloodstream infections were also estimated for each pathogen group (Supplementary File). The number of healthcare-associated bacteraemia/bloodstream infections was estimated for all organisms in both models except for *Staphylococcus aureus *, *Escherichia coli,* and *Klebsiella* spp. which used exact data from the UKHSA [25, 26]. The complete breakdown of the data for these infections is present in the Supplementary File (Table S1, England Model and USA Model).

## Colonizations from antibiotic-resistant organisms

The number of patients colonized with antibiotic-resistant *Staphylococcus aureus, Escherichia coli, Enterococcus faecium, Klebsiella* spp., *Enterobacter* spp. and *Acinetobacter* spp. was based on the lower estimates in different publications [9, 27–29]. The number of colonizations for the other five pathogen groups was not estimated due to the lack of suitable source information [30]. The colonization:infection ratio (Table 1) generated the number of colonizations from resistant organisms (MRSA, VRE, ESBL, CPE and CRAB) for the previously mentioned pathogens relative to the total number of infections for each pathogen group. Omitting the colonization burden altogether would not reflect the true cost of an effective sequencing intervention, as only by proactively sequencing infections and asymptomatic close contacts (such as healthcare workers) can chains of transmission be successfully defined and interrupted [31].

## WGS surveillance and detection of clusters

Estimating the impact of a WGS intervention in a healthcare setting is challenging because there are few published prospective WGS surveillance studies [11, 31]. In SNP-based analysis, suspected transmission events are determined by investigating the number of SNPs that differ between any two isolates [9, 32, 33].

The Cluster Frequency (Table 1) represents the probability of a cluster detected from the total number of isolates for each pathogen group when using WGS, as in the Gordon model. The Cluster Frequency of *Stenotrophomonas maltophilia *, *Pseudomonas* spp., *Citrobacter* spp. and *Serratia* spp. was estimated to be 0.02 based on the lowest estimate of the other modelled organisms for which there is evidence in the Gordon model [9]. The Cluster Frequency of *Clostridiodes difficile* was estimated to be higher, at 0.03, as a previous study suggested 35 % of isolates from a 4 year study were genetically linked by 0 to 2 single nucleotide variants (SNVs) [34].

The Decreased Cluster Size variable (Table 1), was used to determine the proportion of total infections which could be reduced (i.e. the larger this input, the more successful the WGS intervention). Again as *Stenotrophomonas maltophilia *, *Pseudomonas* spp., *Citrobacter* spp. and *Serratia* spp. had not been modelled before, their Decreased Cluster Size (Table 1) estimates were conservatively based on the evidence of other organisms in the Gordon model [9]. The Decreased Cluster Size of *Clostridioides difficile* was higher than these four pathogen groups but not as high as *Escherichia coli, Enterococcus* spp. and *Enterobacter* spp. based on the evidence in the previously mentioned paper from the University of Oxford [34]. The same methodology was used to estimate the reduction in colonizations. The overall reduction in infections and colonizations was estimated based on previous models and sequencing studies [9, 34]; with more effective interventions following more timely and higher resolution analysis, the reduction would be expected to be greater.

In the Gordon model, the turnaround time is 7 days; another prospective study that tried to replicate this had mean turnaround times of 13 days [31]. As the Decreased Cluster Size values in this analysis are based on the Gordon model, this cluster detection and intervention model is also based on a 7 day turnaround time. In addition to the 7 day turnaround time of results, a further assumption is that transmission chains are broken upon identification of outbreak strains, by cleaning wards/rooms, isolating patients or removing equipment. Sensitivity analyses explore differences in Decreased Cluster Size. A turnaround of less than 7 days would be anticipated to increase the speed of detection [12], and thus increase interruption of transmission, but this is not addressed in the model.

## Expected deaths

In addition to the economic modelling, the mortality rate from bacterial HAIs was estimated for each pathogen group by using mortality statistics from the UKHSA [22]. Where national statistics were unavailable, mortality rates from bloodstream infections were used to estimate the number of deaths. For example, *Stenotrophomonas maltophilia* bacteraemia is associated with a mortality rate of 20–60 % [35], and there were 391 *Stenotrophomonas maltophilia* bacteraemia isolates detected in England in 2018 [18]. Conservatively estimating that just under half (47 %) of these *Stenotrophomonas maltophilia* isolates were healthcare-associated [36], the total number of deaths estimated from *Stenotrophomonas maltophilia* HAIs was 37, 20 % of 184. The same methodology was used in both the England and USA models where national statistics were unavailable. The number of deaths was generated as a proportion of the total number of infections and can be seen in the Supplementary File (Table S1, England Model and USA Model).

## Resources and costs

Patients colonized with resistant organisms incurred costs of WGS; the number of patients colonized with CPEs and CRAB incurred the additional costs of PPE and cleaning and nursing time. To avoid over-complicating the model, the cost of nursing and cleaning time were calculated by using the average time spent on an infection or colonization according to the National Institute for Health and Care Excellence (NICE), as the cost of these interactions varies widely [37]. Infected patients accrued the additional costs of antibiotic treatment with associated consumables, infection prevention and control team time and closed bed days from extra length of hospital stay. Antibiotic treatment costs in the UK-based model were determined using current prices as listed in the British National Formulary [38] for England and from the Drugs.com website for the USA [39]. Consumable costs consisted of cannulas, lines, dressing, etc., and were estimated from the length of the antibiotic treatment course and NICE guidelines [37]. Sensitivity analyses were conducted on the cost of all variables in Table 2 except consumables, antibiotic treatment, and the cost of the infection prevention and control team based on available evidence; this was to highlight how fluctuations in these costs would impact the model.

The cost of WGS was the sum of the individual costs of sequencing a bacterial isolate such as the DNA preparation, library preparation, sequencing, analysis and shipping for a total baseline cost of £160 and $194 for England and the USA respectively. The price of library preparation and sequencing is based on the costs quoted by an existing clinical service that provides rapid WGS. Sensitivity analysis was used to explore changes in the cost of sequencing within a wide range of costs [15, 40]. The upper and lower input values can be seen in Table 2 for each variable where there is a range of different prices. The cost of routine microbiological screening was not included as the microbiology test occurs regardless of the sequencing intervention.

## Bed costs, bed days and length of stay

The main cost of an HAI is the extra length of hospital stay [16]. For this reason, conservative estimates for bed-day calculations were used to reduce the possibility of overestimating savings. The length of stay was divided into Colonization Length of Stay and Infection Length of Stay to estimate the amount of PPE used, using the logic of Gordon’s model [9], which can be seen in Table 2. Closed Bed Days from extra length of hospital stay were used to calculate the extra amount of time a patient would occupy a bed if infected, also following the logic in the Gordon model [9]. To account for the difference in the price of a general ward bed and an ICU bed it was estimated that 0.04 % of the HAIs modelled in this study were managed in the ICU [21, 41, 42]. Sensitivity analyses were performed using lower and upper limits of these inputs (Fig. 1, Table 2).

## Cost of clinically qualified specialists in infection prevention and control

By reducing the total number of HAIs, the time an infection prevention and control team would have spent on addressing these infections can be spent elsewhere. This is not a monetary saving but an opportunity cost associated with a reallocation of time. Nevertheless, previous studies have included figures detailing healthcare professionals’ time and cost which are spent on HAIs [10]. Depending on the size of the hospital, infection prevention and control teams will consist of nurses, physicians, public health personnel, epidemiologists and microbiologists, all of varying expertise and salary [43, 44]. The cost of an infection prevention and control team was estimated by taking the number of hospitals in England (1648 [45]); multiplying it by the average annual salary of an individual in an infection prevention and control team in the NHS (average annual salary of individuals in an infection prevention and control team = £64 714.71 – including on-costs [46, 47]); multiplying it by the average number of infection prevention and control professionals per hospital (estimated as 6.6 per 500 bedded hospital [44, 48, 49]), and multiplying that by the amount of time spent on outbreak investigations and HAIs (estimated as 10 % [10]). The same methodology was used to determine the cost of an infection prevention and control team in the USA, using the total number of hospitals in the USA as 6093 [50]; an average salary input of $76 933 [43]; the average number of infection prevention and control professionals per hospital as eight and the time spent on outbreak investigations and HAIs as 10 %, as in Kumar’s economic model [10]. Dividing each of these numbers by the total number of HAIs for each country provides a figure for the cost of an infection prevention and control team per infection of £84.43 and $222.69 for England and the USA.

## Results

The primarily economic model of the impact of diagnostic WGS for surveillance of priority HAI species includes an assessment of its impact on clinical outcomes. The patient-focused outcomes indicate that 393 229 infections and 6073 deaths occur due to bacterial HAIs in England using current epidemiological methods, which is broadly consistent with other published evidence from England which estimates the total number of HAIs (viral, fungal and bacterial combined) to be 653 000 associated with 22 800 associated deaths [21]. WGS-based surveillance, modelled on a turnaround time of 7 days, is predicted to lead to a reduction of 74 408 infections and 1257 deaths. The baseline model estimated a median reduction of total infections and colonizations by 11 % (mean of 18 %). Current practice has a cost to NHS England of around £3 billion (excluding current microbiological screening costs), and the addition of a WGS diagnostic surveillance has an estimated cost of £61.1 million. Based upon the modelled impacts of WGS surveillance, this is associated with an overall saving of £478.3 million.

The USA-based model indicates that 912 141 infections and 30 851 deaths occur from bacterial HAIs based on current epidemiological methods, which is also consistent with published evidence from the USA [24]. WGS-based surveillance, modelled on a turnaround time of 7 days, is predicted to lead to a reduction of 169 260 infections and 4862 deaths. Current practice costs $18.3 billion (without current microbiological screening costs), and the addition of WGS diagnostic surveillance has a cost of $169.2 million. Based on the modelled outcomes of implementing WGS surveillance this is associated with an overall saving of $3.2 billion. The number of bacterial HAIs and deaths for each pathogen group from the current practice and the potential number of avoided infections for both England and the USA are shown in Table 3 and in the Supplementary File (Table S1, the England Model and the USA Model).

**Table 3.** Estimated number of HAIs and deaths for each pathogen group using current practice and estimated number of avoided infections and death with a WGS-based system

	Current practice		WGS	
Organism	HAI	Death	HAI avoided	Death avoided
**England**				
* Staphylococcus aureus *	100 044	1009	10 765	109
* Stenotrophomonas maltophilia *	1842	37	174	3
* Escherichia coli *	73 515	1456	15 071	298
* Enterococcus* species	61 694	1252	25 572	519
* Klebsiella* species	58 359	986	3793	64
* Enterobacter* species	16 074	294	6105	112
* Acinetobacter* species	9171	128	2201	31
* Clostridioides difficile *	41 685	375	7816	70
* Pseudomonas* species (mainly *P. aeruginosa *)	8337	375	787	35
* Citrobacter* species	14 173	68	1338	6
* Serratia* species	8337	92	787	9
Total	393 229	6073	74 408	1257
**USA**				
* Staphylococcus aureus *	202 073	17 176	21 743	1848
* Stenotrophomonas maltophilia *	3722	74	351	7
* Escherichia coli *	148 490	1336	30 440	274
* Enterococcus* species	124 612	2530	51 652	1049
* Klebsiella* species	117 876	2299	7662	149
* Enterobacter* species	32 466	1623	12 331	617
* Acinetobacter* species	18 523	926	4446	222
* Clostridioides difficile *	168 394	2526	31 574	474
*Pseudomonas species* (mainly *P. aeruginosa *)	50 518	1516	4769	143
* Citrobacter* species	28 627	137	2702	13
* Serratia* species	16 839	707	1590	67
Total	9 12 141	30 851	169 260	4862

The full breakdown of each pathogen group’s resistance, species allocation and number of bacteraemia/bloodstream infections is presented in Table S1, the England Model and the USA Model.

As can be seen in Table 3, this clinical impact model estimated *Staphylococcus aureus* to be the most common bacterial HAI in both England and the USA, and most deaths in the USA, with 17 176 deaths annually. In comparison, *Escherichia coli* was responsible for the most nosocomial deaths in England, with 1456 deaths.

Cost per bacteraemia/bloodstream infection was calculated separately by country, sensitivity/resistance and requirement for ICU. By this method a CPE *Klebsiella* spp. bacteraemia was demonstrated to cost £18 904, which is similar to findings reported in a paper from Imperial College, London, which estimated a single outbreak of CPE *Klebsiella* sp. to cost £1 million for 40 CPE *Klebsiella* isolates (£25 000 per infection) [51]. Comparatively, the cost of a single sensitive *Klebsiella* spp. bacteraemia that does not require ICU was estimated to be £6770. In the USA, the cost of a CPE *Klebsiella* spp. bacteraemia requiring ICU was estimated to cost $38 166, whereas its sensitive counterpart not requiring ICU would cost $18 945.

The breakdown of costs for both England and the USA can be seen in Table 4. The largest cost-saving WGS would provide by preventing bacterial HAI would not be direct hospital savings but the opportunity costs associated with the reallocation of existing infrastructure such as hospital beds and healthcare professionals. The complete breakdown of costs associated with each pathogen group can be accessed in the England and USA models in the Supplementary File.

**Table 4.** Estimated differences in costs in England and the USA with current practice and WGS surveillance

	England		USA	
	Current practice	WGS	Current practice	WGS
**Direct hospital resources**				
WGS	£–	£61,118,073	$–	$169,245,276
PPE	£266,750,089	£212,448,937	$740,286,599	$593,475,563
Consumables	£63,113,318	£51,170,819	$177,685,114	$144,713,345
Antbiotic treatment	£319,695,715	£259,060,468	$1,665,639,355	$1,353,822,910
Total cost of hospital resources	£649,559,122	£583,798,298	$2,583,611,068	$2,261,257,094
**Allocation of hospital beds and healthcare professionals**				
Extra length of stay – General Ward	£2,220,992,705	£1,820,127,623	$15,278,211,667	$12,501,524,165
Extra length of stay – ICU	£4,234,642	£3,479,079	$168,858,625	$146,123,367
Cleaning and nursing time	£24,289,160	£19,696,643	$68,180,372	$55,534,825
Cost of infection prevention and control team	£33,200,330	£26,918,060	$203,127,450	$165,434,527
Total cost of allocation of hospital beds and healthcare professionals	£2,282,716,838	£1,870,221,405	$15,718,378,114	$12,868,616,885
Overall total	£2,932,275,960	£2,454,019,702	$18,301,989,182	$15,129,873,979
Overall cost savings with WGS surveillance	£478,256,258		$3,172,115,203	

When using various inputs, the sensitivity analyses demonstrate that overall savings for England and the USA were always retained (Figs 1 and S1). The model was most sensitive to inputs that affected hospital length of stay calculations, such as the number of hospital admissions/inpatients, general ward bed day cost, organism frequency, cluster size decrease and number of closed bed days due to extra length of hospital stay.

The number of colonizations was estimated for the same resistant organisms as in the Gordon model, which estimated a colonization burden for different species to be between 2 and 20 (median=3.2, mean=6.2) times greater than the number of antibiotic-resistant infections in each pathogen group [9]. In the model presented here, the number of colonizations from resistant organisms was made more conservative (especially for VRE, for which colonization was previously estimated at a very high level; detail of revisions is summarized in the Supplementary File, in both the England and USA models) and estimated to between 0.6 and 2.7 (median=1.82, mean=1.77) times greater than the number of antibiotic-resistant infections in each pathogen group. Furthermore, the number of CPE and CRAB colonizations was estimated from the total number of colonizations from resistant organisms to include in PPE calculations (Supplementary File, England and USA models). There is sparse evidence of the prevalence of colonizations from resistant nosocomial organisms, and even less for susceptible organisms; some studies have looked at the colonization of individual organisms in healthcare workers and staff, but only voluntarily, and it has been argued that probable missing links in the transmission chain highlight the need to sequence as many infections and potential colonizations in a hospital as possible [52, 53].

## Discussion

Normally, a new health intervention needs to justify its costs in terms of reduced morbidity and mortality. Proactive diagnostic bacterial genomics is different, in that it improves both patient care and hospital resource management at the same time, in a way that it gives a return on investment: in this model of £7.83 per pound spent in the UK, and $18.74 per dollar in the USA. When scaled, this results in savings of £478.3 million per annum to NHS England and around $3.2 billion in the USA.

Efficiencies of scale, for example using other instruments such as a NextSeq with runs of greater than 80–100 isolates, would reduce the cost of implementation. Marginal savings are also possible from buying a sequencing machine and using it beyond 2 years. However, as indicated by the sensitivity analysis, variations in the costs of sequencing and analysis are not the principal determinants of the savings.

The average excess length of stay estimates vary greatly from 3.9 days with *Escherichia coli* bacteraemia [14] to 16.6 with ESBL *Escherichia coli* infections [9]. The average excess length of stay for all 11 pathogen groups in this model-based analysis is 9.2, similar to a 2019 study which cites between 8.4 and 9.6 days for >50 000 patients [54] and the Guest study which has a mean additional length of ward stay from acquiring an HAI of 9.1 days [21]. The sensitivity analyses demonstrate that calculations involved in length of hospital stay affect the overall financial outcome the most (Figs 1 and S1). The analyses highlight the potential saving if hospital length of stay from these infections on average becomes longer. With ageing populations in both countries, and older age associated with increased hospital length of stay and likelihood of developing an HAI, the proposed savings could be far higher than modelled here in the future [54–56].

As more longitudinal sequencing studies are completed in healthcare settings the previously unrecognized scale of hospital transmission is being uncovered. In Singapore, retrospective WGS of *Enterobacterales* identified 58 groups of isolates that were genomically linked [57]. Furthermore, in two New York City hospitals, retrospective genomic analysis of 197 MRSA bacteraemia isolates identified 33 patients in connected isolate groups differing by ≤ 15 SNVs [58]. These studies clearly showed that WGS led to the identification of transmission events which would have otherwise been undetected.

This study has several limitations. The most important is the dependency of the modelling process on the inclusiveness and quality of the infection frequencies and outcomes as available from hospital-derived and published public health data. As more WGS is carried out, it would be useful for annual epidemiological data to be available through government-funded agencies such as the CDC and UKHSA from a larger spectrum of pathogens than currently exists. The model is expected to be reasonably generalizable across the UK, parts of Europe and North America. The model does not address low- and middle-income country settings. In addition, this analysis does not allow adjustments for comorbidities, different hospital demographics, clinical discipline mix, age or sex, and presents a purely economic model rather than a cost–benefit analysis and thus does not include the use of quality-adjusted life years for the additional patient mortality or morbidities [8]. As the model was initially created from inputs based on England data, the data points used in the USA model are inherently less robust. Also, as the USA healthcare system is decentralized and the demographics across the country vary greatly, the ability of the model to be broken down to predict savings from smaller hospitals is unknown.

This economic analysis indicates that substantial savings as well as positive improvements in clinical outcomes are associated with proactive diagnostic genomics of bacterial pathogens. The largest savings are associated with improved use of healthcare resources, due to avoidance of prolonged patient stays and the ability to use facilities more effectively. These savings were robust and consistent when tested by sensitivity analyses, which showed relatively small impacts from the costs of sequencing and analysis. Finally, the same-year returns on investment indicate savings that more than offset the costs of establishing and delivering WGS-based genomics in clinical settings.

## Supplementary Data

Supplementary material 1
